# The impact of sociodemographic determinants and diabetes type‐2 on oral health outcomes: An analytical cross‐sectional study

**DOI:** 10.1002/cre2.846

**Published:** 2024-01-31

**Authors:** Ashwaq Alkahtani, Paul Anderson, Aylin Baysan

**Affiliations:** ^1^ Institute of Dentistry, Bart's and the London School of Medicine and Dentistry Queen Mary University of London London UK; ^2^ The College of Applied Medical Sciences (CAMS) King Saud University Riyadh Saudi Arabia

**Keywords:** diabetes type 2, oral health, removable prosthesis, sociodemographic determinants

## Abstract

**Objectives:**

This study compared adults with type 2 diabetes (T2DM) and those without diabetes (ND) from East London in terms of sociodemographic characteristics, oral health behaviors, dietary practices, and alcohol and tobacco‐related habits.

**Materials and Methods:**

A total of 182 participants (*n* = 91 for each group) were recruited and requested to complete the validated questionnaire with 33 items.

**Results:**

Results showed that the mean ± SD age was 61 ± 11.7 in the T2DM, while 51 ± 11.2 in the ND group. The mean ± SD age at T2DM diagnosis was 43 ± 10. There was a significant gender difference, with more males in the T2DM group (67.7%) and more females in the ND group (64.8%). Asian‐British (38.4%) were significantly high in the T2DM group when compared to other ethnicities. 92.3% of T2DM participants were significantly more likely to use medications in comparison to the ND group (29.7%). The T2DM participants' personal statements on general health were fair (34%) and good (46.2%) when compared with the ND group (15.4% and 59.3%, respectively). The majority of T2DM and ND participants (98%) lacked dental insurance. In the T2DM group, 31.8% were receiving benefits, and 39.5% were retired, while 46% of the ND group were full‐time employees. Tooth brushing twice a day was slightly less common in T2DM (68%) when compared to the ND group (78%). Nearly half of the participants in both groups failed to carry out interdental cleaning (T2DM = 52%; ND = 47%), and 38.5% of the T2DM group used mouthwash occasionally, while 30% of the ND group had it twice daily. There was a weak association between chewing paan and annual income in ND participants (*r* = .90, *p* = .49). There were significant differences in the presence of removable prostheses, juice, and sweetened juice consumptions between the two groups (*p* < .05).

**Conclusion:**

Within the confines of this study, being male, Asian British, retired due to disability, polypharmacy, and the presence of removable prostheses were all significant factors for T2DM.

## 1 INTRODUCTION

Diabetes mellitus (DM) is one of the major non‐communicable diseases and is considered a threat to global health due to overall healthcare expenditures (Pérez & Smith, 2019). An estimated 415 million individuals are affected by this disease, which is expected to loom to 642 million by 2040 (Ogurtsova et al., 2017). A rise in the prevalence of DM at around 2.5% per year was also reported (Kotwas et al., 2021). Lin et al. (2020) identified diabetes as a leading cause of mortality and decreased life expectancy for individuals worldwide. Elevated levels of proinflammatory mediators in diabetes (especially poorly controlled) would play a role in the elevated risk of oral diseases such as periodontal destructions, xerostomia, and dental caries. Therefore, oral health might also have an impact on type 2 diabetes mellitus (T2DM) risk and metabolic control, which is a complex bidirectional link between diabetes and oral diseases (Al‐Lawati et al., 2002; Watt et al., 2019).

Kyrou et al. (2020) conducted a study on the sociodemographic and lifestyle‐related risk factors for groups of individuals with T2DM in Europe. These authors reported many risk factors related to the development of T2DM, such as age, ethnicity, family history, low socioeconomic status, education level, obesity/being overweight, metabolic syndrome, specific unhealthy lifestyle practices, and emotional factors, i.e., stress, anxiety, and depression emerged. Firstly, the risk factors for increased body weight with a lack of physical activities play an essential role in patients with T2DM. Secondly, a few metabolic syndrome components, that is, hypertension, are related to T2DM. Thirdly, individuals' unhealthy eating and dietary habits, such as the high intake of processed meat, sugar‐sweetened beverages, and low consumption of fruits and vegetables are major concerns. Finally, cigarette and tobacco smoking were also deemed as another vital factor. Therefore, it was noted that there is a need for targeted health programs to be implemented so that these risk factors can be controlled to manage T2DM. Meanwhile, Fletcher et al. (2002) discussed that risk factors related to genetics/environment, and metabolism are interconnected and led to the advancement of T2DM disease.

In this respect, Kotwas et al. (2021) noted the importance of patient education as a priority. However, the content of patient education needs to be tailored according to the demographics of patients as well as their DM history, background, and general health. Government policies also need to be implemented to improve healthcare access for patients living with diabetes to enhance the quality of life. With T2DM seen as a leading disease that shortens individuals' lifespans with compromised quality of life, systems and institutions would need to start working together. This collaboration could focus on preventing and providing optimum health policies.

Interestingly, Leite et al. (2013) assessed oral health and its association with T2DM. It was reported that inflammation is the key to this association. In this respect, the involvement of oral healthcare professionals in identifying individuals at risk of diabetes would provide a clear screening process and preventive strategies to slow down the development of T2DM and related oral complications. However, there is still a lack of awareness of the possible association between oral health factors and T2DM, both for patients and health professionals. Therefore, the investigation and examination of various factors that impact the behaviors and characteristics of individuals with T2DM could enable health professionals and policymakers to understand the scope of the problem.

In this respect, a study using the QDScore (Hippisley‐Cox et al., 2009) to predict the development of T2DM in East London revealed that at least 20% of individuals are at high risk of developing the disease within the next decade. The risk mapping revealed the “East London Diabetes Belt,” where 1 in 10 people have a high risk of developing T2DM, rising to approximately one in six in East London, UK (Mathur et al., 2012). Therefore, the aim of this questionnaire‐based case–control study was to assess and compare the sociodemographic characteristics, oral health behaviors, dietary habits, and alcohol and tobacco‐related habits among T2DM and ND individuals in East London, UK.

## 2 METHODOLOGY

### 2.1 Study design

This analytical cross‐sectional study was conducted between December 2020 and November 2021. Before the study, ethical approval was obtained from the Office of Research Ethics Committees (REC reference number: 08/H0702/54).

An overall sample size of 91 individuals (182 in total) was proposed to assess the extent of dental caries between the groups, with a power of 80% and a significance level of .05. This calculation assumed that 50% and 30% of individuals in the case and control groups, respectively, would have carious lesions, which was considered the minimum clinically relevant difference. A total of 400 patients were screened. 182 T2DM and ND participants (*n* = 91 for each group) were recruited for this case‐control study according to inclusion and exclusion criteria (Table 1). Subsequently, participants were requested to sign the written consent forms before completing the questionnaire.

**Table 1:** Inclusion and exclusion criteria.

Inclusion criteria	Exclusion criteria
− Participants who are male or female ≥ 18 years of age. − For the test group, they have been diagnosed with type 2 Diabetes − For the control group, participants who are not diagnosed with type 2 Diabetes − Participants having a minimum of six natural tooth − They are capable of giving informed consent − They have the ability to understand and speak English − They are able and willing to comply with all trial requirements − They are not participating in another dental trial − They are not diagnosed with cognitive defects due to mental illness, depression, Alzheimer's disease, or dementia. − No antibiotics, no steroidal and/or nonsteroidal anti‐inflammatory medication used during the last 3 weeks − Participants who are not pregnant and also not breastfeeding − Participants who are not in another dental study testing different dental products during the previous three months and during the study period − Participants who are not currently taking Vitamin D supplements	− Participants who are edentulous − Cognitive defect due to mental illness, depression, Alzheimer's disease, or dementia. − The presence of any hard or soft tissue tumors in the oral cavity − Patients undergoing chemo and/or radiation therapy − Any other significant disease or disorder which, in the opinion of the Investigator, may either put the participants at risk because of participation in the trial or may influence the result of the trial or the participant's ability to participate in the trial. − Any condition, which in the opinion of the investigator, would preclude participation by the subject (such as cross‐infection control risk) − Participants who are prescribed long‐term systematic antibiotics − Participants who are pregnant and breastfeeding − Participants who are in another dental study testing different dental products during the previous three months and during the study period − Participants who had additional fluoride treatment in the past 6/3 months − Participants who are prescribed to use highly fluoridated toothpaste − Participants who are currently taking Vitamin D supplements

### 2.2 Questionnaire structure

The questionnaire comprised 33 items pertaining to sociodemographic factors, oral health behaviors, and dietary, alcohol, and tobacco‐related habits. According to Del Greco et al. (1987), such questionnaires are required to be consistent and precise when measuring the data and variables. Therefore, the widely validated questions from a previous study (Yuen et al., 2009) were adopted and modified. The resulting questionnaire was reviewed by three experts in the field *prior* to the application for ethical approval and according to the suggestions, modifications were carried out. Subsequently, the questionnaire was sent to the members of the UK‐based Diabetes Patient lay groups (*n* = 8) to assess its reliability, and the questions were again modified according to the responses of these participants.

This final questionnaire contained 33 items covered by two sets of covariates.

#### 2.2.1 Sociodemographics and self‐reported general health

The first set of questions in the questionnaire focused on the participants' sociodemographic factors, including gender, ethnicity, marital status, education attainment, employment status, income/benefits, dental insurance, medication use, general health, and household income.

#### 2.2.2 Social and oral health status with dietary attitudes

The second set of questions was divided into three components: the participants' behaviors related to social and oral health status with dietary attitudes. Tooth brushing frequency/time, using dental floss, mouthwash use, frequency of dental check‐up visits, and the presence of removable prosthesis were recorded. Habits related to drinking alcohol, using chewing pan, and smoking tobacco were noted. Questions included diet and frequency of the variety of drinks consumed, fruit and vegetable intake, and the number of snacks daily.

### 2.3 Data analysis

The distributions of the study variables by T2DM status were reported as means and standard deviation (SD) for continuous and as number (percentage) for categorical variables. The descriptive and inferential statistical analyses were carried out using the IBM Statistical Package for the Social Sciences (SPSS® Statistics, V21.0 software; International Business Machines Corporation). Data were identified according to the range or absolute numbers with percentages using this software. Pearson's and Chi‐squared tests for associations and proportions were also performed at 95% confidence intervals. Differences below the 5% limit (*p* < 0.05) were considered significant.

## 3 RESULTS

### 3.1 Sociodemographics and self‐reported general health

**Figure 1:**
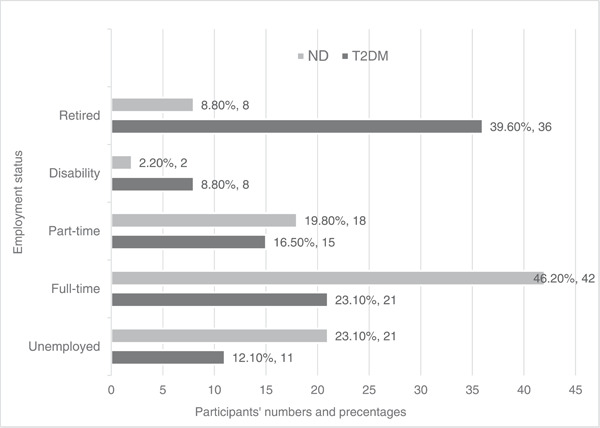
Employment status for both groups (*p* < .0005). ND, without diabetes; T2DM, type 2 diabetes mellitus.

Table 2 shows the sociodemographic characteristics of the study population. There was a significant gender difference, with more males in the T2DM (67.7%) and more females in the ND group (64.8%). Asian British were significantly more in the T2DM (38.4%) when compared to other ethnicities. In addition, participants with T2DM were retired (39.6%) and reported disability (8.8%) when compared to the nondiabetes group (8.8% and 2.2%, respectively). 46.2% of ND participants were full‐time employees (Figure 1), whereas only 23.1% in the T2DM group.

**Table 2:** Breakdown of the participants' sociodemographics for T2DM and ND groups.

Variables	T2DM	ND	*p*‐Value
	(*n* %)	(*n* %)	
Gender			
Male	61 (67.7)	32 (35.2)	<0.001**
Female	30 (32.3)	59 (64.8)	
Ethnicity			
Asian British	35 (38.4)	11 (12.1)	<0.001**
African British	16 (17.5)	26 (28.6)	
East African Asian	1 (1.1)	0 (0)	
White Caucasian	26 (28.5)	15 (16.5)	
South American	1 (1.1)	0 (0)	
Others	12 (13.1)	39 (42.9)	
Marital status			
Married	71 (78)	75 (82.4)	.527
Divorced	12 (13.2)	7 (7.7)	
Single	7 (7.7)	8 (8.8)	
Widowed	1 (1.1)	1 (1.1)	
Educational attainment			
Uneducated	9 (9.9)	4 (4.4)	.625
UG	16 (17.6)	12 (13.2)	
PG	3 (3.3)	15 (16.5)	
Diploma	15 (16.5)	21 (23.1)	
Certificate	48 (52.7)	39 (42.9)	
Employment			
Unemployed	11 (12.1)	21 (23.1)	<.001**
Full‐time	21 (23.1)	42 (46.2)	
Part‐time	15 (16.5)	18 (19.8)	
Disability	8 (8.8)	2 (2.2)	
Retired	36 (39.6)	8 (8.8)	
On benefits			
Yes	33 (36.3)	25 (27.5)	.205
No	58 (63.7)	66 (72.5)	
Dental insurance			
Yes	2 (2.2)	2 (2.2)	1.00
No	89 (97.8)	89 (97.8)	
Medication use			
Yes	84 (92.3)	27 (29.7)	<.001**
No	7 (7.7)	64 (70.3)	
General health			
Poor	8 (8.8)	1 (1.1)	.001
Very Poor	2 (2.2)	3 (3.3)	
Fair	31 (34.1)	14 (15.4)	
Good	42 (46.2)	54 (59.3)	
Very Good	8 (8.8)	19 (20.9)	
Annual Income			
Below £10,000	26 (28.5)	21 (23.1)	.082
£10,000–14,999	16 (17.58)	9 (9.9)	
£20,000–24,999	4 (4.4)	8 (8.8)	
£25,000–29,999	19 (20.88)	18 (19.8)	
£30,000–34,999	10 (11)	10 (11)	
£35,000–39,999	4 (4.4)	2 (2.2)	
£40,000 or 49,999	2 (2.2)	6 (6.6)	
£50,000 or more	9 (9.9)	17 (18.7)	

Abbreviations: ND, without diabetes; T2DM, type 2 diabetes mellitus.

92.3% of T2DM participants were significantly more likely to use multiple medications in comparison wit the ND group (29.7% only). Interestingly, the T2DM participants' personal statements on general health were fair (34%) and good (46.2%) when compared to the ND group (15.4% and 59.3%, respectively). The mean ± SD age was 61 ± 11.7 for the T2DM and 51 ± 11.2 for the ND group. The median and mean (± SD) age at T2DM diagnosis were 45 and 43 (±10) years. The majority of the participants (31.9%) were diagnosed between 55 and 65 years old, while the minority (2.2%) was between 25 and 35 years old. The mean and median duration of diabetes were 18 and 16 years, respectively. In addition, according to the self‐reported diabetes control, 43% reported being in good, whereas 10% stated poor control.

### 3.2 Social and oral health status

Table 3 presents the social and oral health characteristics of the study population. Interestingly, there was a significant difference in relation to the presence of removable prostheses between the T2DM (*n* = 22, 24.2%) and ND participants (*n* = 7, 6.6%) (*p* = .002).

**Table 3:** Breakdown of the study participants' oral health behaviors.

Variables	T2DM *n* (%)	ND *n* (%)	*p*‐Value
Tooth brushing frequency			
Once a day	29 (31.9)	20 (22)	.134
Twice a day	62 (68.1)	71 (78)	
Tooth brushing time			
Morning	18 (19.8)	6 (6.60)	.163
Evening	4 (4.4)	10 (11)	
Morning & Evening	69 (75.8)	75 (82.4)	
Interdental cleaning			
Never	48 (52.7)	43 (47.3)	.163
Occasionally	2 (2.2)	4 (4.4)	
Once	28 (30.8)	36 (39.6)	
Twice	13 (14.3)	8 (8.8)	
Mouthwash use			
No	27 (29.70	24 (26.4)	.101
Occasionally	35 (38.5)	22 (24.2)	
Once	9 (9.9)	17 (18.7)	
Twice	20 (22)	28 (30.8)	
Dental check‐up frequency			
Never	1 (1.1)	29 (31.90)	.653
<12 months	28 (30.80	38 (41.8)	
Once a year	41 (45.1)	20 (22)	
When needed	21 (23.1)	4 (4.4)	
Smoking			
Yes	7 (7.7)	4 (4.4)	.352
No	84 (92.3)	87 (65.6)	
Chewing pan			
Yes	2 (2.2)	4 (4.4)	.408
No	89 (97.8)	87 (95.6)	
Drinking alcohol			
Yes	29 (31.9)	18 (19.8)	.063
No	62 (68.1)	73 (80.2)	
Removable prostheses			
Yes	22 (24.1)	7 (7.6)	.002
No	69 (75.8)	84 (92.3)	

Abbreviations: ND, without diabetes; T2DM, type 2 diabetes mellitus.

Most of the T2DM (*n* = 84; 92.3%) and ND (*n* = 87; 96%) participants were non‐smokers. In addition, 97.8% of the T2DM (*n* = 89) and 95.6% of the ND (*n* = 87) participants did not chew pan. With regard to drinking alcohol, a total of 29 T2DM participants (32%) reported “yes,” which was relatively high, however, insignificantly different (*p* = .63) in comparison to the ND group (*n* = 18,19.8%). Most of the T2DM participants (*n* = 62, 68.1%) brushed their teeth with fluoridated toothpaste twice daily. Meanwhile, a total of 71 ND participants (78%) reported tooth brushing twice a day. In addition, 75.8% of the T2DM (*n* = 69) and 82.4% of the ND participants (*n* = 75) brushed their teeth in the morning and evening. Almost half of the participants in both groups answered “never” when asked about interdental cleaning habits (T2DM = 52.7%; ND = 47.3%). 39% of T2DM participants (*n* = 35) occasionally used fluoridated mouthwash, while 31% of the ND group (*n* = 28) reported the use of mouthwash twice daily. Interestingly, 45.1% of the T2DM group (*n* = 41) indicated dental check‐ups once a year when compared to the ND participants (*n* = 20, 22%).

In addition, there was also a weak association between chewing paan and annual income in the ND participants (*r* = .90, *p* = .049). However, there were insignificant associations either between T2DM or ND participants, and all included oral health variables (*p* > .05).

### 3.3 Dietary attitude

**Figure 2:**
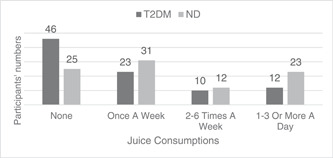
Juice consumptions for both groups (*p* = .002). ND, without diabetes; T2DM, type 2 diabetes mellitus.

A significant difference was reported in juice drink consumption; 50.5% (*n* = 46) of the T2DM participants reported avoiding juice, while only 27.5% (*n* = 25) of the ND group (Figure 2). In addition, most T2DM reported no consumption of sweetened drinks (*n* = 72, 79%) when compared to the ND groups (*n* = 44, 48.4%).

Regarding water uptake, 49.5% (*n* = 45) of the T2DM participants reported drinking three to six glasses of water daily, relatively similar to the nondiabetes group (*n* = 46, 50.5%). Nearly half of the participants in both groups reported no milk consumption (T2DM *n* = 39, 42.9%; ND *n* = 36, 38.6%). Furthermore, 72.5% of both groups stated that they do not take or consume carbonated drinks. The majority of the participants in both groups also opted out of drinking sports beverages (*n* = 86, 94.5%; *n* = 80, 88%, respectively).

Both T2DM and ND participants (*n* = 31, 34%; *n* = 37, 41% respectively) reported consuming snacks one to three times a day. Interestingly, daily consumption of vegetables was similar for the T2DM and ND groups (*n* = 32, 35%; *n* = 29, 32%, respectively). In addition, a total of 44 T2DM participants (48%) reported consuming fruits two to six times a week when compared to the ND group (*n* = 51; 56%), which was slightly high, however insignificant.

## 4 DISCUSSION

This is the first case‐control study in East London to assess and compare sociodemographic characteristics, oral health behaviors with alcohol and tobacco‐related habits, and dietary attitudes among people with T2DM.

The data aligns with previous reports that males account for a large share of the diabetes population, suggesting a significant difference between the sexes in this disease. With few notable exceptions, such as in the Middle East and North Africa, diabetes is more commonly diagnosed in middle‐aged men in comparison to women (Zhou et al., 2016). According to the results from the US National Health and Nutrition Examination Survey, men are at a higher risk of developing diabetes than women (Peters et al., 2019).

British Asian participants constituted the largest ethnic group of people with T2DM in this study. The high prevalence of diabetes in Asia has previously been documented (Sun et al., 2022). Multiple factors, including ethnicity, race, and genetics, have been linked to the development of T2DM (Das & Elbein, 2006). Age, obesity, family history of diabetes, a sedentary lifestyle, and the prevalence of other modern lifestyle disorders such as hypertension and dyslipidemia were linked to an increased risk of developing diabetes (Sameer et al., 2020).

There were substantial variations in both overall health and medication usage for the T2DM group in this study. These results might have a valid point since people living with diabetes are more likely to have other related conditions such as obesity, hypertension, and chronic kidney disease, all of which might contribute to increased health issues and polypharmacy (Saljoughian, 2019). T2DM participants have a 50%–90% increased risk for several domains of disability (Gregg & Menke, 2018) in accordance with this present case‐control study's findings. The percentage of disabled people in the diabetes group was four times more like to be seen in comparison to the nondiabetic group. In addition, as the duration of diabetes and comorbidity increases, it was previously reported that patients require multiple medications for glycemic control (Polonsky et al., 2011). According to Inci's study (2021), 77.9% (473) of diabetes patients used five or more medications. Furthermore, other studies reported a range between 56.5% and 57.1% (Noale et al., 2016; Silva et al., 2018).

Annual income level was not a factor in relation to having T2DM in the study. Contrary to the current outcome, studies demonstrated an association between T2DM and socioeconomic factors. However, the evidence strongly pointed to age, race, family history, socioeconomic class, level of education, and dietary habits/practices as the primary factors leading to the high risk of T2DM pathogenesis, which was strongly related to the impact and relationship of each risk indicator (Fletcher et al., 2002; Kyrou et al., 2020). It has been reported that people with low income were disproportionately affected by the diabetes epidemic (Fleischer et al., 2016; Hwang & Shon, 2014; Rabi et al., 2006). With this respect, the duration of diabetes and age at diagnosis, both of which are associated with metabolic control, might be crucial variables to account for this insignificant correlation. In this study, nearly half of the participants with T2DM presented with relatively self‐reported adequate glycemic control, and the mean age at diagnosis was 43 years old, which is comparable to the Centre for Disease Control and Prevention's (CDC) finding that T2DM developed mostly over the age of 45 (CDC, 2021). Furthermore, the participants had an average of 18 years of disease duration; therefore, it could be speculated that their coping strategies were developed in accordance with their socioeconomic status. Consequently, this might account for the insignificance association between risk factors and oral health. In addition, the complexity and scale of this chronic disease might fail to address the vital underlying risk factors, i.e., socioeconomic status, deprivation, and psychosocial stress in this case–control study. Hence, further research with a diverse background of individuals is required to understand the risk factors related to oral health in people living with T2DM.

The statistical analysis showed the presence of removable prostheses to be significant in T2DM participants (*p* < .05). This could reflect more tooth loss within the T2DM participants in comparison to the ND group. It is important to investigate and explore further the reasons and explanations of this potential association between the presence of removable prosthesis and T2DM. This particular finding was also noted in the study by Lee et al. (2019). The authors identified the presence of removable dental prostheses as a potential risk indicator for the onset of uncontrolled diabetes in Korean men. The authors also revealed that people with removable dentures could potentially have insufficient nutritional intake and might be at an elevated risk for diabetes. These dietary changes may lead to nutritional deficiencies and be associated with the onset of metabolic syndrome and inadequate glycemic control. In addition, people living with diabetes might present with oral complications such as periodontitis, xerostomia, oral infection, and dental caries, which would potentially increase the risk of losing teeth. In this respect, Mayard‐Pons et al. (2015) reported that people with diabetes tend to undergo dental extractions early and often when compared to individuals without diabetes. Furthermore, individuals living with diabetes might also be provided with removable prostheses instead of dental implants due to substantial bone loss, which could be related to a history of periodontal diseases. It should be noted that there is clear evidence of the bidirectional relationship between diabetes mellitus and periodontitis (Preshaw et al., 2012).

In addition, nearly 80% of the T2DM while nearly half of the ND participants opted out of consuming juices and sweetened drinks, which would be expected due to the low sugar diet among people living with diabetes. It was also reported that reducing the consumption of sugar‐sweetened beverages has been identified as one of the most cost‐effective prevention and control measures for diabetes (Imamura et al., 2015; Xu et al., 2018). These results were in agreement with Bleich and Wang (2011), and the authors indicated less sugar‐sweetened beverages consumption by diabetes patients.

This study had limitations due to the closed‐ended nature of the questions in the survey. The participants were not provided a platform to explain and expand their perceptions and experiences when answering the questionnaire. However, the open‐ended answers could have provided explanations of the investigated phenomenon and explored participants' responses further. Therefore, performing a mixed method approach where the quantitative data could be supported by the qualitative findings would be considered in future research.

Further research is required to take into account the socioeconomic and socioecological determinants of prediabetes and T2DM from diverse backgrounds to understand the potential oral health complications further in people living with diabetes. The ultimate aim is to detect and manage oral complications and/or diabetes early for optimum quality of life and to save National resources.

## 5 CONCLUSION

Within the limitations of this study, the comparison of T2DM and ND groups enabled the identification of oral health risk factors associated with type 2 diabetes. This study demonstrated that within the East London study population, being male, Asian British, retired due to disability, polypharmacy, and the presence of removable prostheses were all significant factors in people living with T2DM in East London. It is also necessary to consider the interaction of these factors for general and oral health when providing diabetes prevention management. However, further research is required to identify whether these different factors constitute a cluster of interrelated risk behaviors that might be amenable to interventions aimed at reducing the burden of disease attributable to diabetes.

## AUTHOR CONTRIBUTIONS

Aylin Baysan and Ashwaq Alkahtani were involved in the conception, design, and conduct of the study and the analysis and interpretation of the results. Ashwaq Alkahtani wrote the first draft of the manuscript, and all authors edited, reviewed, and approved the final version of the manuscript. Aylin Baysan and Ashwaq Alkahtani are the guarantors of this work and, as such, had full access to all the data in the study and take responsibility for the integrity of the data and the accuracy of the data analysis.

## CONFLICT OF INTEREST STATEMENT

The authors declare no conflict of interest.

## ETHICS STATEMENT

The ethical approval was obtained from the Office of Research Ethics Committees (REC reference number: 08/H0702/54). Consent for publication has been obtained.

## Data Availability

Data will be available upon publication.
